# Optimization of an Ultra-Sonication Extraction Method for Major Compounds Found in *Mondia whitei* Using Design of Experiment

**DOI:** 10.3390/molecules27092836

**Published:** 2022-04-29

**Authors:** Ramakwala Christinah Chokwe, Simiso Dube, Mathew Muzi Nindi

**Affiliations:** 1 Department of Chemistry, The Science Campus, College of Science Engineering and Technology, University of South Africa, Corner Christiaan de Wet and Pioneer Avenue, Florida Park, Roodepoort 1709, South Africa; dubes@unisa.ac.za; 2 Institute for Nanotechnology and Water Sustainability, College of Science Engineering and Technology, University of South Africa, Corner Christiaan de Wet and Pioneer Avenue, Florida Park, Roodepoort 1709, South Africa

**Keywords:** *Mondia whitei*, extraction, ultra-sonication, descriptive screening design, central composite design

## Abstract

Optimum extraction conditions are vital in quality control methods to enable accurate quantification of the compounds of interest. An ultra-sonication method was developed for the extraction of seven major compounds found in *Mondia whitei*. Extraction temperature, time, power, frequency, percentage of ethanol in water and solvent to sample ratio were screened to access their significance on the percentage recovery of the compounds of interest. These parameters were screened using Descriptive screening design. Extraction temperature, solvent to sample ratio and the interaction between temperature and percentage ethanol in water were found to have a significant effect on the response. These parameters were then optimized using central composite design. The optimum conditions were found to be 66.1% ethanol in water, 70 °C temperature and 3 mL: 5 mg solvent to sample ratio. This method was successfully applied in the development of a quality control method for the seven compounds in *Mondia whitei* samples.

## 1. Introduction

*Mondia whitei* is a medicinal plant that is native to Africa. It is distributed across different parts of the continent and is therefore, known by various names such as Ogomo in Kenya, Limte in Cameroon, Mulondo in Uganda, and Umondi in South Africa, and in Zimbabwe, they call it Mungurawu [1]. It has been used for many years and still continue to be used traditionally to cure various ailments. The roots are the commonly used part of this plant. They are used to treat aches and pains, hypertension, stress, improve appetite, libido, fertility treatment, mental disorder, diabetes, asthma among other ailments [2,3,4,5]. Above all, the plant is popularly known and used as an aphrodisiac across the whole African continent [6]. The aphrodisiac activity of the roots of the plant has been proven scientifically by several researchers [6,7,8,9].

The popularity of *Mondia whitei* has necessitated the need to isolate and characterized compounds from the plant. This is so that the compounds responsible for the activities can be identified and to also enable quality control of the plant samples that are sold commercially. Most scientific work done on the plant so far was to corroborate the traditional medicinal use of the plant. A few researchers have reported on the isolation and characterization of compounds from the plant. Koorbanally et al., (2008), Mukonyi and Ndiege (2001) isolated 2-hydroxy-4-methoxybenzaldehyde and 3-hydroxy-4-methoxybenzaldehyde from the roots of the plant [10,11,12]. The authors reported that the compound 2-hydroxy-4-methoxybenzaldehyde was responsible for the taste modifying property of the plant. Wang J et al., (2010) reported its antimicrobial and antioxidant activities [13]. Patnam et al., (2004) isolated and identified 6-methoxy-7-hydroxycoumarin and 6-methoxy-7,8-dihydroxycoumarin from the roots [10]. These compounds were found to have antimicrobial activity by Yang et al., (2017) [14].

Ultra-sonic assisted extraction is a low-cost and efficient method whereby extraction can be carried out in short periods of time [15]. This makes it a suitable method for extraction of plant materials. Extraction yield and therefore recovery is known to be affected by factors such as the type of extracting solvent, temperature, ratio of solvent to sample and extraction time [16]. Therefore, it is of paramount importance that the extraction method is optimized for optimum extraction of the compounds of interest. Usually, a one-factor-at-a-time method is used to optimize the factors that are known to influence the response. In this method, one factor is optimized at a time while the other factors are kept constant. Moreover, this method does not consider the interaction between the factors. Therefore, design of experiment can be used as an alternative, since the method not only considers interactions between factors, but the number of experiments is fewer as compared to one-factor-at-a-time, especially when many factors are being investigated [17]. 

In this study, an extraction method was optimized for the extraction of 2-hydroxy-4-methoxybenzaldehyde (C1), 3-hydroxy-4-methoxybenzaldehyde (C2), 2,4-dihydroxy-6-methylbenzaldehyde (C3), 7-hydroxy-6-methoxycoumarin (C4), 7,8-dihydroxy-6-methoxycoumarin (C5), coumarin (C6) and phenantherene (C7) which have been identified in *Mondia whitei*. The optimum extraction conditions are not only important for maximum extraction of the compounds of interest but also to enable accurate quantification of the compounds in *Mondia whitei* products, thereby enabling quality control of *Mondia whitei* samples. In the design of the experiment, descriptive screening design has an advantage over other screening methods because it requires fewer experiments and the factors are accessed using three levels [18]. In this study, factors which were identified as being significant using descriptive screening design were further optimized using central composite design and response surface methodology. Furthermore, an HPLC-DAD method was used to separate and analyze the extracts. The optimum extraction method for these compounds has been used successfully for their extraction and subsequent accurate quantification in *Mondia whitei* samples and or products [19]. 

## 2. Results and Discussion

### 2.1. Screening of the Extraction Factors Using Descriptive Screening Design

**Figure 1:**
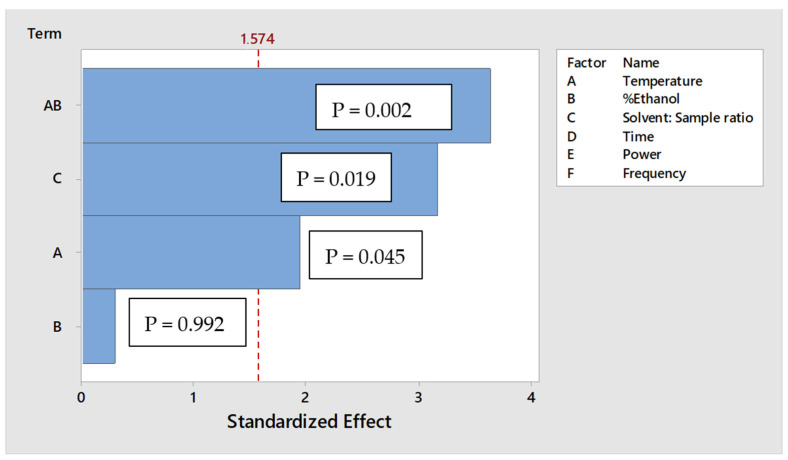
Pareto chart showing the effects of the critical quality parameters on the response.

Temperature, ratio of ethanol to water, ratio of solvent to sample, extraction time, sonication power and frequency are known to affect the percentage recovery of compounds from plants materials. Therefore, these factors were selected as the independent variables and were screened for their effect on the percentage recovery of the compounds of interest using ultra-sonication technique. The percentage recovery of the compounds was identified as the dependent variable. Descriptive screening design (DSD) has the advantage over other screening methods because less experiments are required for the same number of factors and main effects are not cofounded with two-factor interactions. Therefore, DSD is recommended if the number of independent variables is more than four [20]. The experiments generated using the Minitab software as well as the responses that were obtained experimentally are shown in Table 1, the experiments were randomized to avoid biases. An average percentage recovery of the compounds was chosen as the response because it was necessary to find optimum extraction conditions for simultaneous extracts of the compounds of interest. Stepwise selection was used to assess the significance of the factors on the response. The significant effects of the factors are shown in the pareto chart (Figure 1), temperature, the ratio of solvent to sample and the interaction between temperature and percentage ethanol in water had a significant effect on the percentage recovery of the compounds. Time, power, frequency and the other interactions were not significant as shown by their absences from the pareto chart. Even though percentage of ethanol in water does not have a significant effect on the response, it was included due to hierarchy, since its interaction with temperature is significant.

Only the significant factors were chosen to be optimized. 

### 2.2. Optimization of the Extraction Factors Using Central Composite Design (CCD)

**Figure 2:**
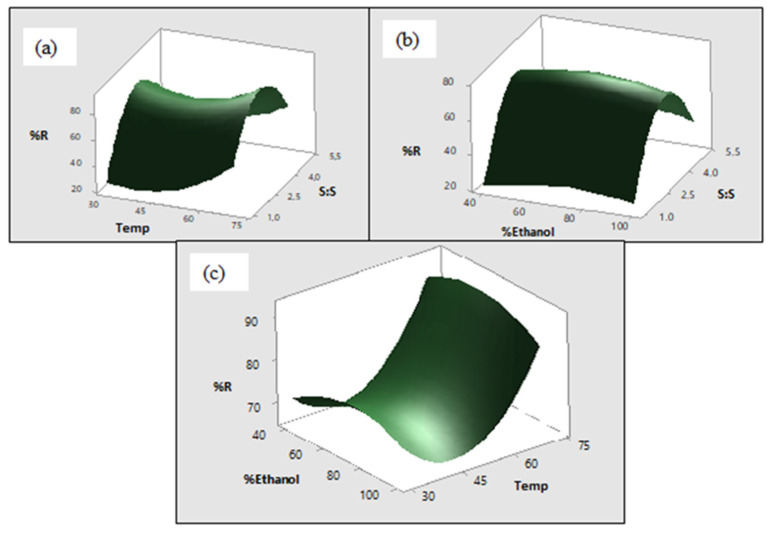
Surface and contour plots showing percentage recovery as function of the effects temperature and solvent ratio (S:S) while keeping percentage ethanol constant (**a**); effects of percentage ethanol and solvent ratio (S:S) while keeping temperature constant (**b**); and effects of percentage ethanol and temperature while keeping solvent ratio constant (**c**).

CCD was used for optimization of the significant factors identified by the screening method. Extraction time, power and frequency were kept constant at 20 min, 0.03 watts and high frequency respectively while optimizing the significant factors. The experiments generated by the Minitab software together with the response are given in Table 2. R^2^ and R^2^ adjusted for the model are given in Table 3, these were 90.10 and 81.20% respectively. These parameters indicate how well the model predict the response and descriptive ability of the model respectively [21]. The linear and second-order models were significant with *p*-values of 0.016 and 0.000, respectively (Table 3). The two-way interaction model was not significant shown by *p* > 0.05. The non-significant value of lack of fit with (*p* = 0.969) indicated that the model fits well with the experimental design and can be used to predict the response. Modelling of the response was done using second-order polynomial as shown in Section 3.4. The surface and contour plots were used to visualize the results. The surface plots are curved because the model has second order terms that are statistically significant (Figure 2). The full model that includes non-significant factors is given in Equation (1) below.

Response = 72.07 − 0.02x + 7.76y+ 8.26z − 4.51x^2^ + 12.39y^2^ − 35.95z^2^ − 1.14xy − 1.34xz − 6.02yz (1)

where x = %ethanol; y = extraction temperature, z = solvent: sample ratio.

#### 2.2.1. Effects of Solvent: Sample Ratio

**Figure 3:**
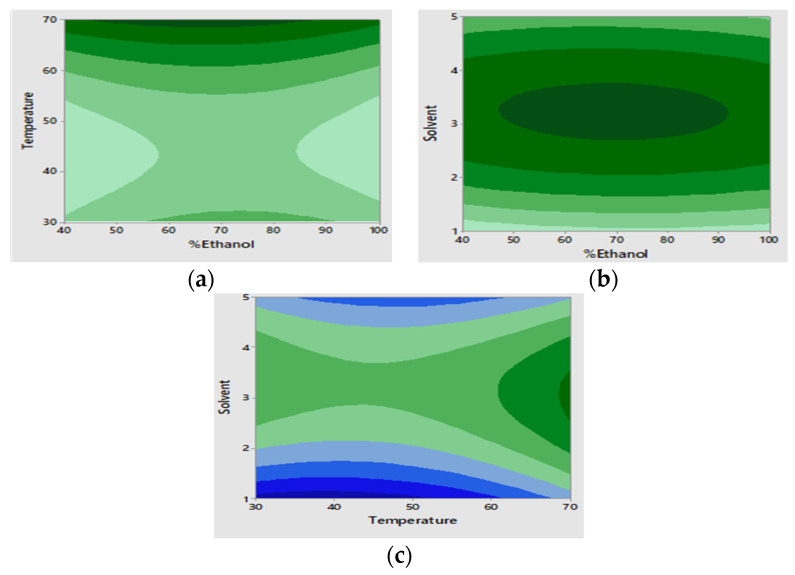
Surface and contour plots showing percentage recovery as function of the effects temperature and solvent ratio while keeping percentage ethanol constant (**a**); effects of percentage ethanol and solvent ratio while keeping temperature constant (**b**); and effects of percentage ethanol and temperature while keeping solvent ratio constant (**c**).

This was found to be the most significant factor/variable with *p* = 0.013; the effect of this factor on the response was positive as shown by its positive regression coefficient. The surface plots (Figure 2a,b) show that the percentage recovery increases as the solvent: sample ratio increases up to 4 mL: 50 mg, this could be attributed to the mass transfer between the solid material and the solvent due to the difference in concentration gradient [22]. Above 4 mL: 50 mg the percentage recovery decreases with an increase in solvent: sample ratio. A similar trend was observed by Chen et al., (2020) in their investigation for the effects of solvent: sample ratio on the yield of phytochemicals from coffee leaves [16]. The contour plots show that the optimum solvent is between 2.7 mL and 3.8 mL, shown by the dark green regions in Figure 3b,c.

#### 2.2.2. Effects of Temperature

An increase in temperature is known to result in an increase in percentage recovery [21,22]. In this study, temperature had a significant effect on the response (*p* = 0.018 coefficient = 7.76). However, the effect of temperature on the response was the opposite up to 45 °C as shown by the surface plot thereafter it increases with an increase in temperature. The contour plots show that optimum extraction can be obtained with temperature from 68.55 °C, indicated by the dark green region (Figure 3a,c).

#### 2.2.3. Effects of %Ethanol in Water

Different ratios of ethanol: water have been reported for the extraction of the compounds from *Mondia whitei*. Therefore, it was necessary to optimize this factor. Percentage ethanol in water had no significant effect on the response in this study, as shown by *p* > 0.05 for this factor. This is also observed on the surface plots, where no change in response is observed when percentage ethanol increases. The contour plots show that the optimum percentage ethanol in water is between 46.6 and 91.5%.

### 2.3. Testing the Predicted Optimum Conditions

**Figure 4:**
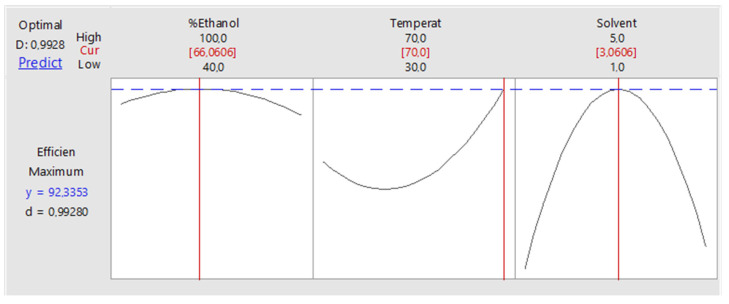
The optimum conditions predicted using the Minitab v18 software (State College, PA, USA).

**Figure 5:**
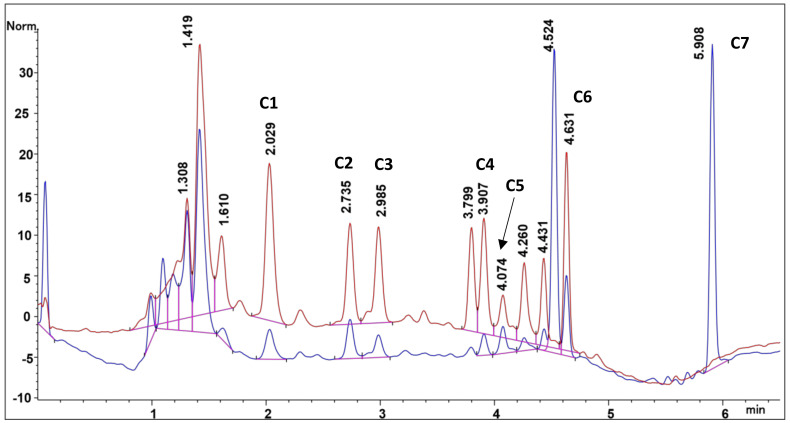
Chromatogram representing the separation of the Mondia whitei roots powder spiked with the compounds of interest under optimised conditions by gradient elution mode; at 0 min 25% B, 2 min 40% B, 3 min 65% B, 4 min 100% B with a run time of 6.5 min. Injection volume of 5 µL, flow rate of 1.3 mL min^−1^ and temperature of 25 °C.

The optimizer in the Minitab software was used to predict the optimum conditions for each independent factor (Figure 4). The setting for the independent factors was so that they should be maximized. The optimum conditions that were predicted are percentage ethanol (66.1%), temperature (70 °C) and solvent to sample ratio (3 mL: 50 mg) and the predicted desirability functions was 0.9928. These conditions were tested by extracting the compounds of interest under the predicted optimum conditions. The results that were obtained were used to calculate the desirability function. The practical and predicted desirability functions were compared and the percentage difference was calculated. The calculated desirability function was 0.9773, comparing that with the predicted desirability function, the percentage difference is 0.02% which shows that both the experimental and predicted values are in agreement. This method was applied successfully to extract the compounds of interest from *Mondia whitei* samples and products (Figure 5) in turn enabling their accurate quantification [19]. Figure 5 shows a chromatogram representing separation of the *Mondia whitei* roots powder which was spiked with the compounds of interest then extracted using the developed extraction method. The separation conditions are shown in the caption for Figure 5.

## 3. Materials and Methods

### 3.1. Chemicals and Material

The solvents that were used in this work were of analytical grade with a purity of >95%, they were purchased from Sigma-Aldrich (Steinheim, Germany). The standards were also purchased from Sigma-Aldrich. The aqueous solutions were prepared using ultra-high-purity (UHP) water (18.2 mΩ) from a Milli-Q water purification system (Molsheim, France) and filtered using a 0.45 µm membrane filter Sigma-Aldrich (Steinheim, Germany). The plant material was obtained from Durban, Kwazulu-Natal in South Africa and was authenticated at the college of Agriculture and Environmental Sciences (University of South Africa, Florida Park, Roodepoort, South Africa). The roots were grinded using a kitchen blender after which the powder was separated from the fibers using 0.25 mm mesh Sieve.

### 3.2. Instrumentation

**Figure 6:**
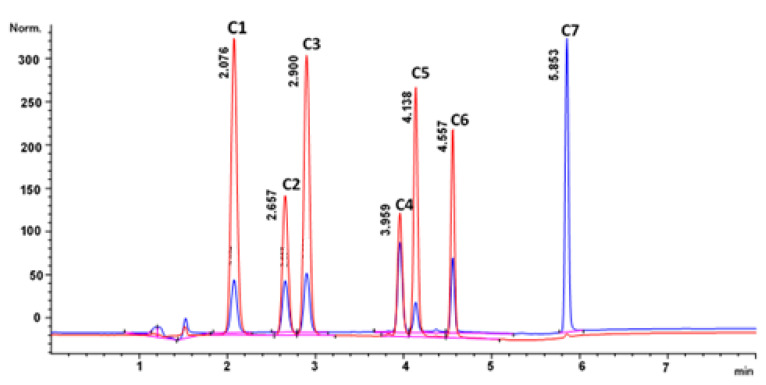
Typical chromatogram of separation of the C 1–7 under optimised conditions by gradient elution mode; at 0 min 25% B, 2 min 40% B, 3 min 65% B, 4 min 100% B with a run time of 6.5 min. Injection volume of 5 µL, flow rate of 1.3 mL min^−1^ and temperature of 25 °C.

Ultrasonic-assisted extraction was performed using an ultrasonic bath (ScientTech, Labotec, Midrand, South Africa). Separation of the compounds was performed using an Agilent HPLC 1260 system (Agilent Technologies, Waldbronn, Germany) which consisted of a binary high-pressure pump, autosampler, a thermostatted column compartment, a diode array detector and a fluorescence detector. Instrument control, data collection and processing were achieved using the ChemStation (version 1.9.0) software. The separation of the mixture was performed on an XTerra^®^ MS C18 (150 mm × 4.6 mm, 3.5 µm) analytical column (Waters Corporation, Milford, MA, USA). The mobile phase used for the separation was 0.1% formic acid in water (A) and acetonitrile (B). The following gradient elution mode was used to separate the compounds: 0 min 25% (B), 1 min 35% (B), 2 min 45% (B), 3 min 55% (B), 5 min 100% (B). Injection volume was 5 µL; temperature was 25 °C; and flow rate was 1.3 mL min ^−1^. The compounds were monitored at 254 and 331 nm. A typical chromatogram for the separation of the pure standards in the solvent is shown in Figure 6.

### 3.3. Preparation of the Samples for Extraction

The stock solutions of the standards were prepared at a concentration of 500 mg L^−1^, by diluting 2.5 mg of each standard with ethanol in 5 mL volumetric flasks. Fifty milligrams of the *Mondia whitei* roots powder was spiked with 200 µL of each standard and extracted under the experimental conditions shown in Table 1. After extraction, the extracts were separated from the undissolved materials using a centrifuge (Eppendorf, Hamburg, Germany) at 3000 rpm for 6 min. The extracts were dried using a freeze dryer (BioBase, Shandong, China). The dried extracts were then reconstituted with 1 mL of ethanol in preparation for HPLC-DAD analyses.

### 3.4. Experimental Design

Descriptive screening design was used to assess the significance of the following independent factors: Extraction temperature; ratio of ethanol: water, solvent: sample ratio, extraction time, ultrasound power and frequency on the percentage recovery of the seven compounds of interest. Percentage recovery of the compounds were calculated using Equation (2).

% Recovery = Peak area _found_ − peak area _original_/peak area _spiked_ (2)

where peak area found is the peak area of the compounds found in the spiked sample after extraction, peak area original is the peak area of the compound found in the unspiked sample after extraction, peak area spiked the peak area of the spiked compound in the pure solvent.

The design consisted of six factors, three level, two centre points and fourteen experiments. Table 1 shows the minimum and maximum values for each factor. The experiments were randomized to avoid systemic error (Table 4). The Central Composite Design consisted of three factors, three levels, six centre points and six axial points with twenty experiments (Table 2). The experiments were run in triplicates. Analysis of variance was used to assess the effect of the factors on the response. Response surface methodology was used to visualize the results in the form of surface and contour plots. The response was analyzed using a second-order polynomial regression equation as follows:

(3) $$Y=\beta_{o}+\sum_{i=1}^{n}\beta_{1}x_{1}+\sum_{i=1}^{k}\beta_{11}x_{1}^{2}+\sum_{1}^{k-1}\beta_{12}x_{1}x_{2}.$$

where Y is the response, β_o_; β_1_; β_11_ and β_12_ are the intercept, linear, quadratic and interaction regression coefficients, respectively.

### 3.5. Software

Minitab Version 18 from Minitab Inc. (State College, PA, USA) was used for the creation of the experimental design, statistical and graphical analysis.

## 4. Conclusions

A simple and fast extraction method was developed and optimized for seven major compounds found in *Mondia whitei*. Extraction temperature and the ratio of solvent to sample were found to have a statistically significant effect on the percentage recovery of the compounds of interest. The optimum conditions for extraction of these compounds were 66.1% ethanol in water, 70 °C temperature and 3 mL to 5 mg sample. The developed method can be used to extract the compounds of interest from *Mondia whitei* products to enable their quantification.

## Figures and Tables

**Table 1:** Descriptive screening design experiments and the response.

Run Order	1	2	3	4	5	6	7	8	9	10	11	12	13	14
Temperature	70	30	70	50	50	50	30	30	30	70	70	30	70	50
Ethanol ratio	100	100	40	70	100	70	70	40	40	40	100	100	70	40
Solvent volume	3	5	4	3	5	3	1	3	5	1	1	1	5	5
Time	60	20	40	40	60	40	60	20	60	20	20	40	20	60
Power	0.01	0.03	0.05	0.03	0.05	0.03	0.05	0.05	0.01	0.05	0.05	0.01	0.01	0.01
Frequency	Low	Low	Low	High	High	High	Low	High	Low	Low	Low	High	High	High
% Average recovery	44.58	53.53	72.91	46.50	42.03	65.98	55.92	45.22	31.67	73.72	66.41	68.34	44.88	58.85

**Table 2:** CCD experimental conditions and response for optimization of the extraction method.

Run Order	% Ethanol	Temperature	Solvent: Sample Ratio	% Average Recovery
1	40	50	3	68.62
2	100	70	1	49.99
3	100	30	5	47.67
4	100	30	1	27.05
5	40	30	5	50.17
6	70	50	1	22.52
7	40	70	1	51.70
8	70	50	5	80.85
9	40	30	1	19.58
10	100	70	5	51.15
11	70	50	3	78.24
12	70	70	3	92.86
13	70	50	3	78.99
14	70	50	3	82.18
15	40	70	5	53.58
16	70	50	3	76.57
17	70	30	3	77.17
18	100	50	3	67.63
19	70	50	3	57.54
20	70	50	3	56.67

**Table 3:** ANOVA results for optimization of the extraction method using CCD.

	*p*-Value	Coefficient
Linear	0.016	
Square	0.000	
2-way interaction	0.303	
Constant	0.000	72.07
%Ethanol	0.996	−0.002
Temperature	0.018	7.76
Solvent	0.013	8.26
%Ethanol*%Ethanol	0.410	4.51
Temperature*Temperature	0.040	12.39
Solvent*Solvent	0.000	−35.95
%Ethanol*Temperature	0.719	−1.14
%Ethanol*Solvent	0.673	−1.34
Temperature*Solvent	0.079	−6.02
Lack-of-fit	0.969	
R^2^	90.10%	
R^2^(adj)	81.20%	
R^2^(pred)	76.65%	

**Table 4:** The independent factors and their labels in uncoded form.

	Temperature	Ethanol Ratio	Solvent Volume	Time	Power	Frequency
Low	30	40	1	20	0.01	Low
High	70	100	5	60	0.05	High

## Data Availability

The data presented in this study is available on request from the corresponding authors.

## Sample Availability

Samples of the compounds used in this study, are available from the authors.

## References

[1] South African National Biodiversity. http://pza.sanbi.org/mondia-whitei.

[2] Oketch-Rabah H.A. (2012). *Mondia whitei*, a medicinal plant from Africa with aphrodisiac and antidepressant properties: A review. J. Diet Suppl..

[3] Aremu A.O., Cheesman L., Finnie J.F., Van Staden J. (2011). *Mondia whitei* (Apocynaceae): A review of its biological activities, conservation strategies and economic potential. S. Afr. J. Bot..

[4] Balogun F.O., Tshabalala N.T., Ashafa A.O. (2016). Antidiabetic Medicinal Plants Used by the Basotho Tribe of Eastern Free State: A Review. J. Diabetes Res..

[5] Patrick-Iwuanyanwu K.C., Nkpaa K.W. (2015). Toxicity Effect of Sub-Chronic Oral Administration of Class Bitters^®^-A Polyherbal Formula on Serum Electrolytes and Hematological Indices in Male Wistar Albino Rats. J. Xenobiot..

[6] Watcho P., Zelefack F., Ngouela S., Nguelefack T.B., Ngouela S., Telefo P.B., Kamtchouing P., Tsamo E., Kamanyi A. (2007). Effects of the Aqueous and Hexane Extracts of *Mondia whitei* o sexual behaviour and some fertility parameters of sexually inexperienced male rats. Afr. J. Tradit. CAM.

[7] Gundidza A., Mmbengwa G.M., Magwa V.M., Ramalivhana M.L., Mukwevho N.J., Ndaradzi N.T., Samie W. (2009). Aphrodisiac properties of some Zimbabwean medicinal plants formulations. Afr. J. Biotechnol..

[8] Watcho P., Donfack M.M., Zelefack F., Telesphore A., Nguelefack B., Kamtchouing W.S., Tsamo P., Kamanyi E. (2005). Effects of the hexane extract of *Mondia whitei* on the reproductive organs of male rats. Afr. J. Tradit. CAM.

[9] Quasie O., Martey O.N.K., Nyarko A.K., Gbewonyo W.S.K., Okine L.K.N. (2010). Modulation of penile erection in rabbits by *Mondia whitei*: Possible mechanism of action. Afr. J. Tradit. CAM.

[10] Patnam R., Kadali S.S., Koumaglo K.H., Roy R. (2005). A chlorinated coumarinolignan from the African medicinal plant, *Mondia whitei*. Phytochemistry.

[11] Koorbanally N.A., Mulholland D.A., Crouch N.R. (2008). Isolation of Isovanillin from aromatic roots of the medicinal African Liane *Mondia whitei*. J. Herbs Spices Med. Plants.

[12] Mukonyi I., Ndiege K.W. (2001). 2-hydroxy-4-methoxybenzaldehyde: Aromatic taste modifying compound from *Mondia whytei* skeels. Bull. Chem. Soc. Ethiop..

[13] Wang J., Zhao J., Gao H., Zhou L., Liu Z., Chen Y., Sui P. (2010). Antimicrobial and antioxidant activities of the root bark essential oil of Periploca sepium and its main component 2-hydroxy-4-methoxybenzaldehyde. Molecules.

[14] Yang J.Y., Park J.H., Lee M.J., Lee J.H., Lee H.S. (2017). Antimicrobial Effects of 7,8-Dihydroxy-6-Methoxycoumarin and 7-Hydroxy-6-Methoxycoumarin Analogues against Foodborne Pathogens and the Antimicrobial Mechanisms Associated with Membrane Permeability. J. Food Prot..

[15] Izadiyan P., Hemmateenejad B. (2016). Multi-response optimization of factors affecting ultrasonic assisted extraction from Iranian basil using central composite design. Food Chem..

[16] Chen X., Ding J., Ji D., He S., Ma H. (2020). Optimization of ultrasonic-assisted extraction conditions for bioactive components from coffee leaves using the Taguchi design and response surface methodology. J. Food Sci..

[17] Wahid Z., Nadir N. (2013). Improvement of One Factor at a Time Through Design of Experiments 1. World Appl. Sci. J..

[18] Jones B., Nachtsheim C.J. (2013). Definitive screening designs with added two-level categorical factors. J. Qual. Technol..

[19] Chokwe R.C., Dube S., Nindi M.M. (2020). Development of a Quantitative Method for Analysis of compounds found in *Mondia whitei* using HPLC-DAD. Molecules.

[20] Hocharoen L., Noppiboon S., Kitsubun P. (2020). Process Characterization by Definitive Screening Design Approach on DNA Vaccine Production. Bioeng. Biotechnol..

[21] Sulaiman I.S.C., Basri M., Masoumi H.R.F., Chee W.J., Efliza S. (2017). Effects of temperature, time, and solvent ratio on the extraction of phenolic compounds and the anti-radical activity of Clinacanthus nutans Lindau leaves by response surface methodology. Chem. Cent. J..

[22] Belwal T., Pandey A., Bhatt I.D., Rawal R.S. (2020). Optimized microwave assisted extraction (MAE) of alkaloids and polyphenols from Berberis roots using multiple-component analysis. Sci. Rep..

